# Supplementary material for: Characterization of the development of the mouse cochlear epithelium at the single cell level
Source: Nat Commun. 2020 May 13;11:2389. doi: 10.1038/s41467-020-16113-y (PMC7221106; doi:10.1038/s41467-020-16113-y)
Supplement: Supplementary file 1 — Supplementary Information [file 41467_2020_16113_MOESM1_ESM.pdf]

Characterization of the development of the mouse cochlear epithelium at the single cell level

Kolla et al.

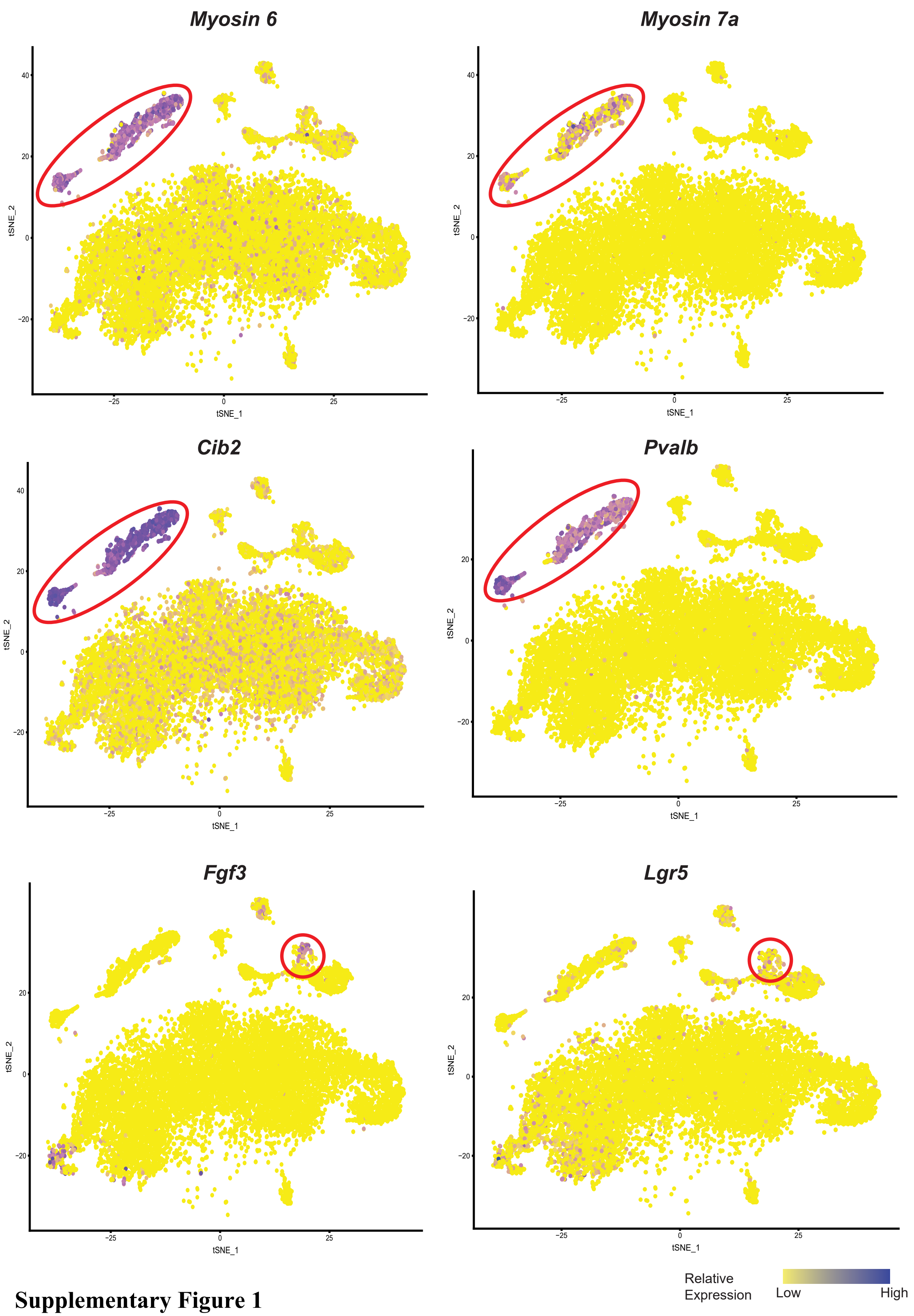

Supplementary Figure 1

Feature plots illustrating hair cell and supporting cell markers in P1 cells

Hair cell (*Myo6, Myo7a, Pvalb* and *Cib2*) and 3rd Row Deiters' cell (*Fgf3, Lgr5*) markers are indicated by red circles.

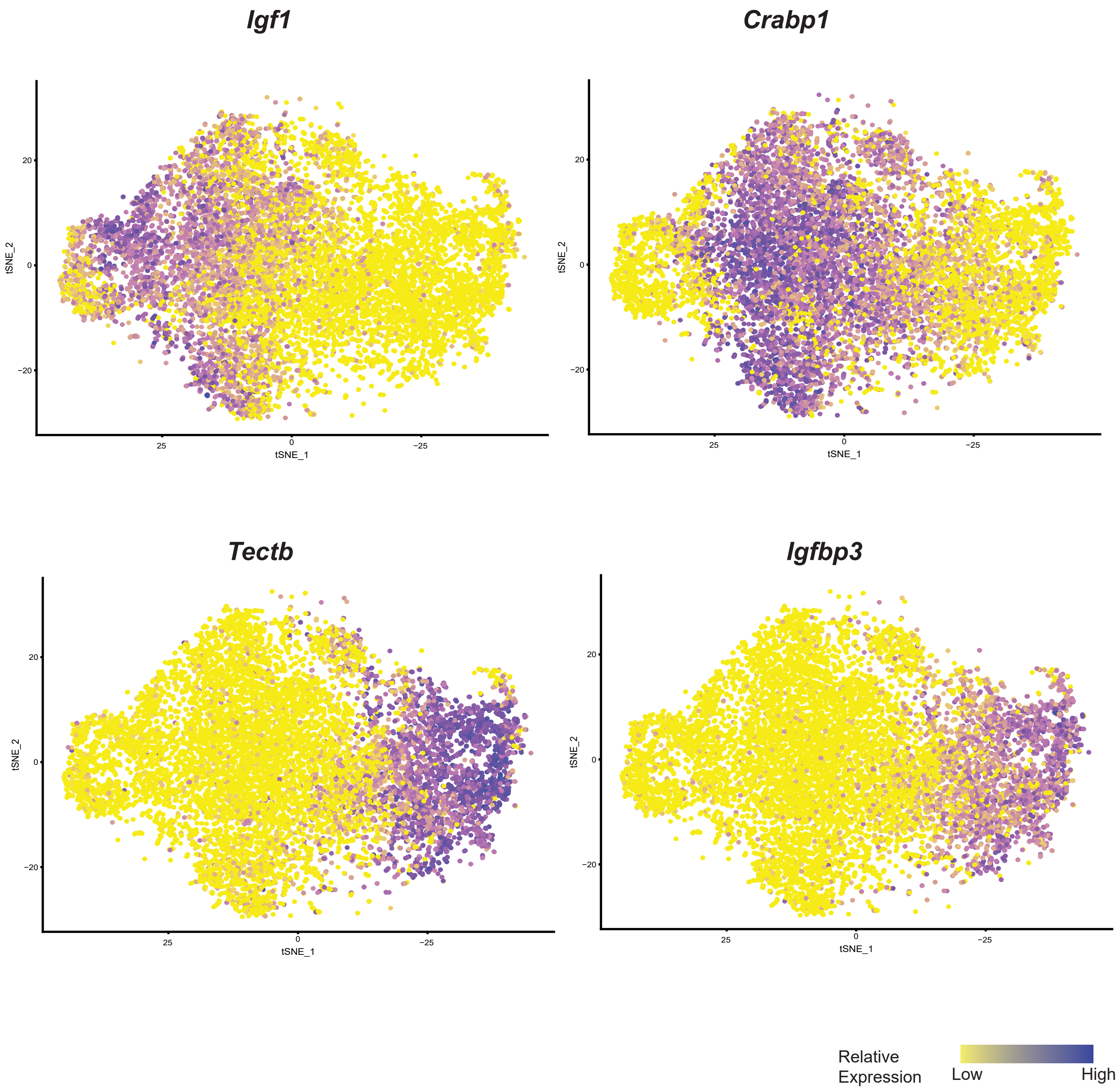

Supplementary Figure 2

Feature plots for gene markers in different regions of Kölliker's organ

Note that each marker is expressed in a band of cells along the tSNE axis.

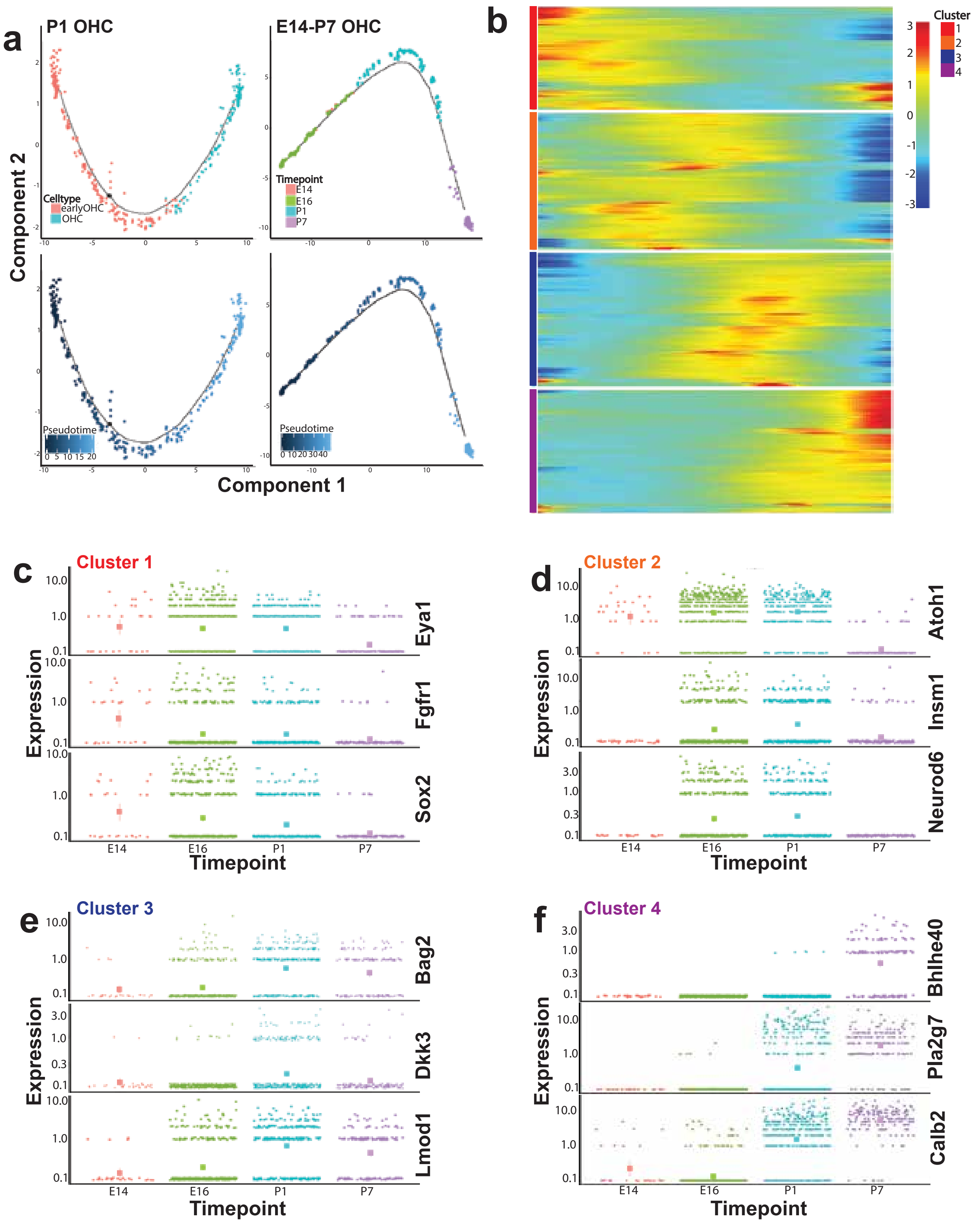

Supplementary Figure 3

Monocle analysis for all OHCs from either P1 alone or E14, E16, P1 and P7 combined
a. Cells are arranged by identity (for P1 alone) or by age of collection. Bottom graphs show position along the pseudotime line. For both data sets, the trajectory mirrors normal development b. Heatmap of gene expression for all OHC along pseudotime. Color codes match the four developmental phases described for P1 OHCs, see Figure 3. Also see Supplementary Data 5 for a complete list of genes in each phase. C-F. Jitter plots illustratin g expression level of the indicated gene in each individual cell for OHCs from each time poin t. Representative genes from each of the four developmental clusters are illustrated.

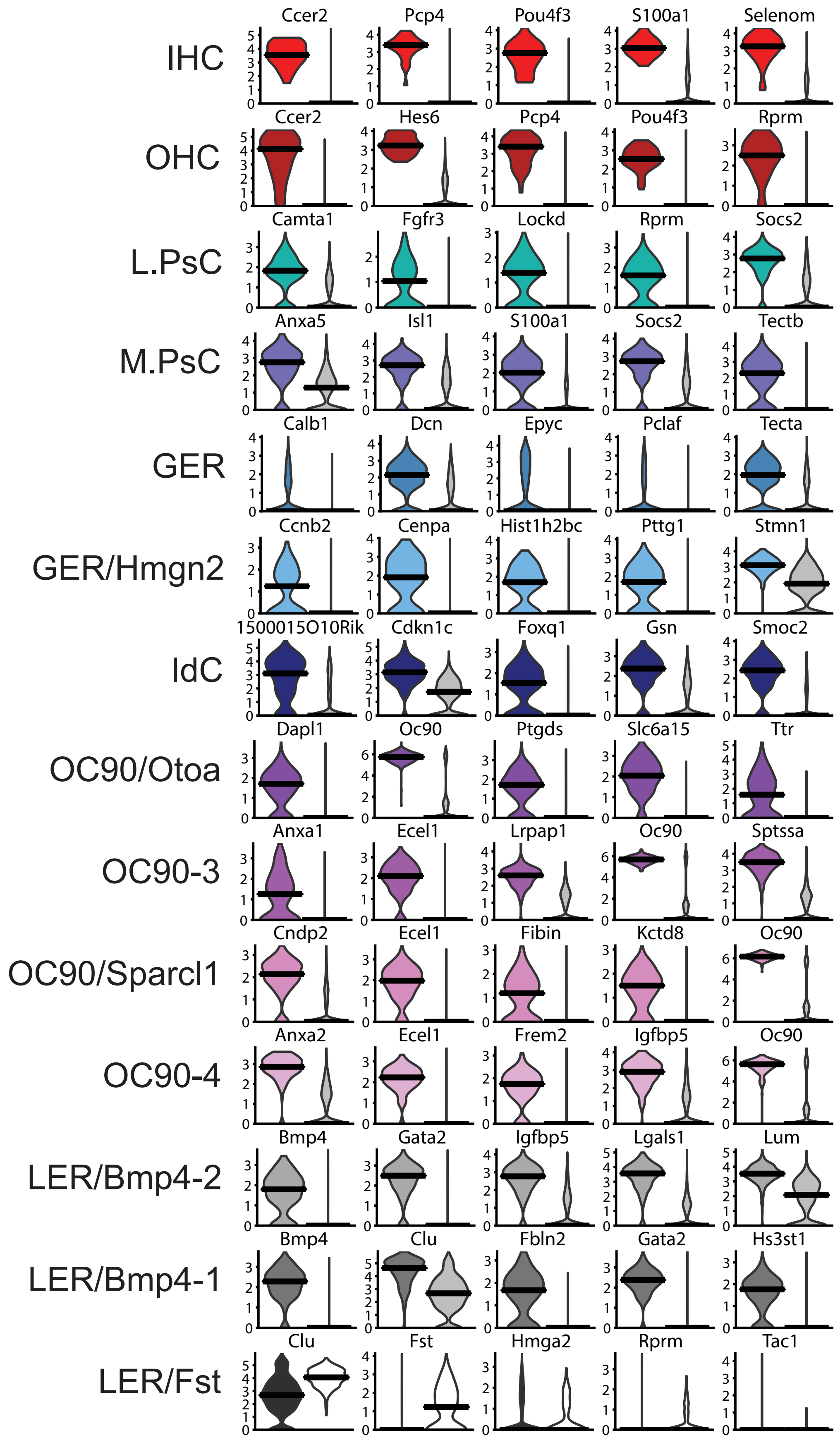

Supplementary Figure 4

Differentially expressed genes in E14 cochlear cells

Top 5 differentially expressed genes for each cell cluster in the E14 data set.

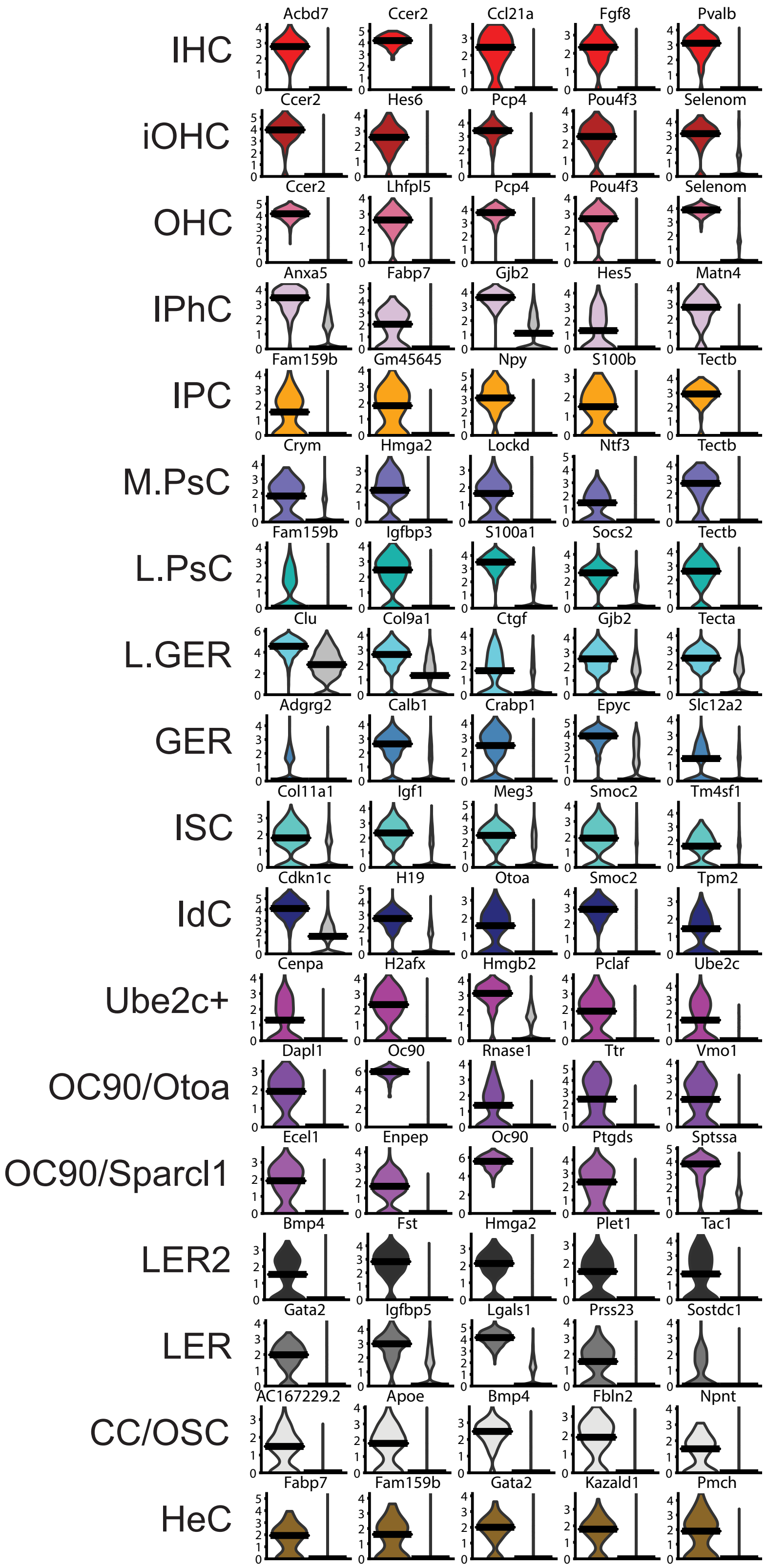

Supplementary Figure 5

Differentially expressed genes in E16 cochlear cells

Top 5 differentially expressed genes for each cell cluster in the E16 data set.

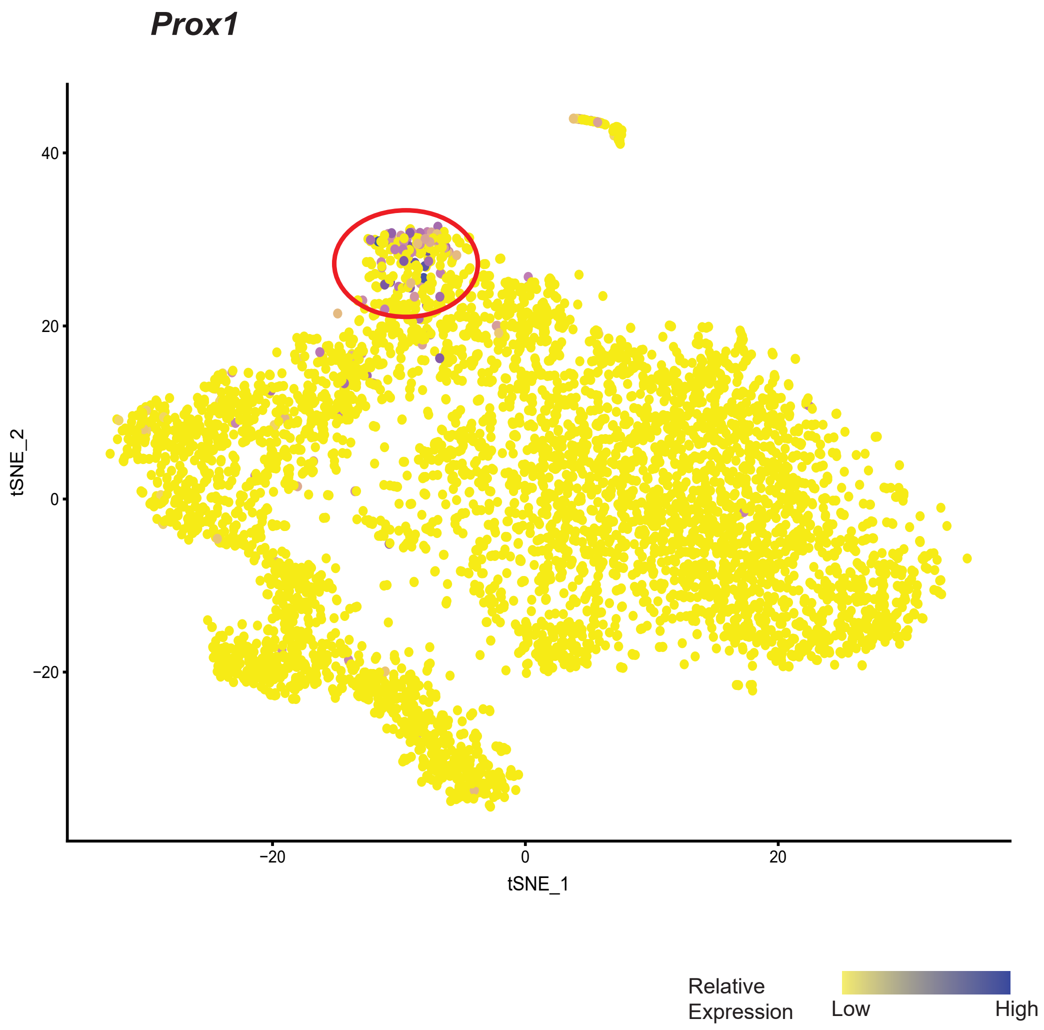

Supplementary Figure 6

Feature plot illustrating cells with high expression of *Prox1* in the E14 data set

Expression overlaps with *Fgfr3,* see Figure 4.

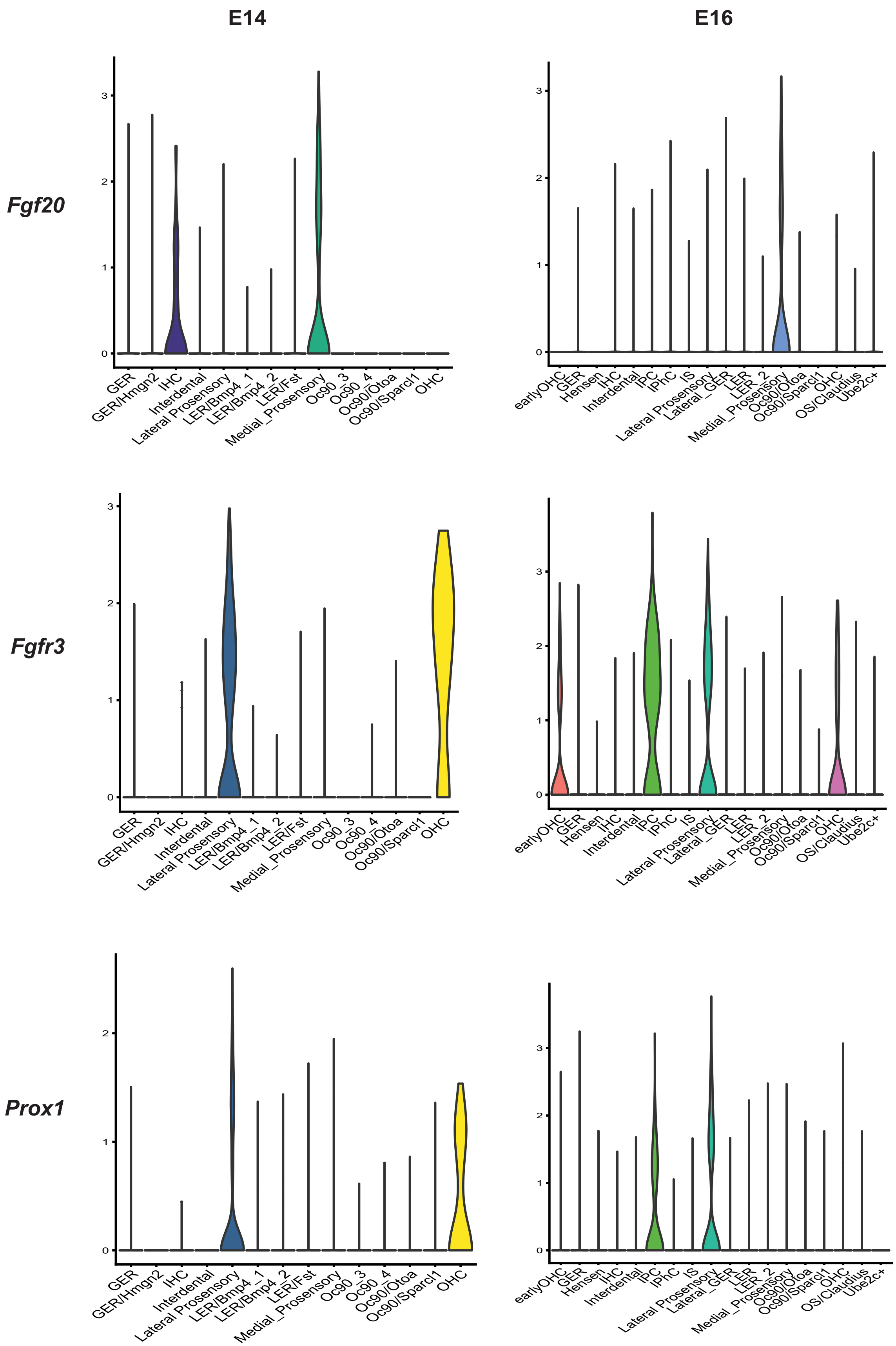

Supplementary Figure 7

Expression of lateral genes in cochlear cells at E14 and E16

Violin plots for *Fgf20, Fgfr3* and *Prox1* in E14 and E16 data sets. Note that *Fgf20* is restricted to MPsCs and IHCs at E14 and only to MPsCs at E16. *Fgfr3* and *Prox1* are restricted to LPsCs and OHCs at E14 and to LPsCs, IPCs and OHCs at E16.

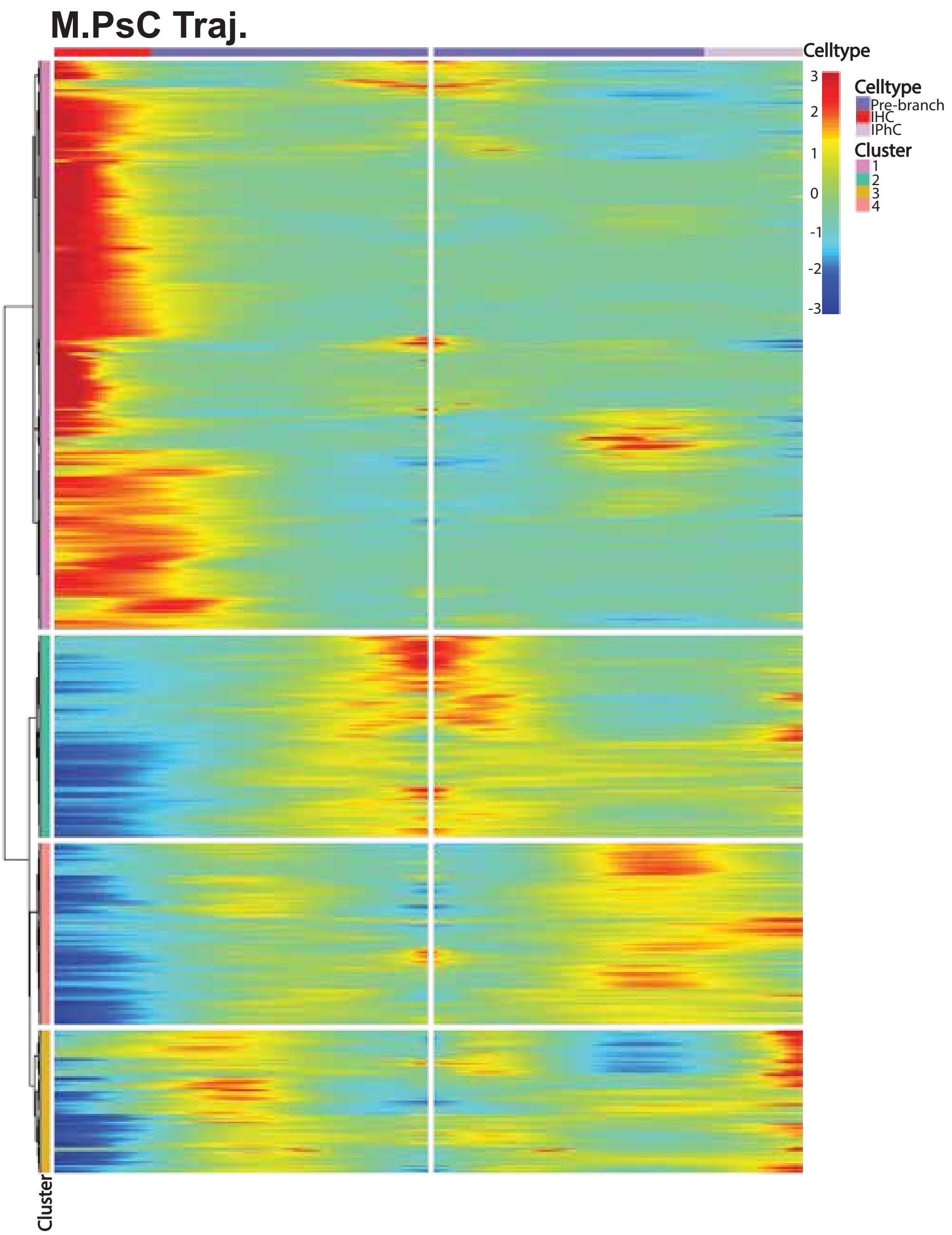

Supplementary Figure 8

Trajectory analysis for development of inner hair cells and inner phalangeal cells

Pseudotime heatmap illustrating changes in gene expression along the two branches of the medial prosensory timeline. Center of the heatmap indicates prosensory cells. Inner hair cells are to the left while Inner Phalangeal cells are to the right. See Supplementary Data 10 for lists of genes in each of the developmental clusters.

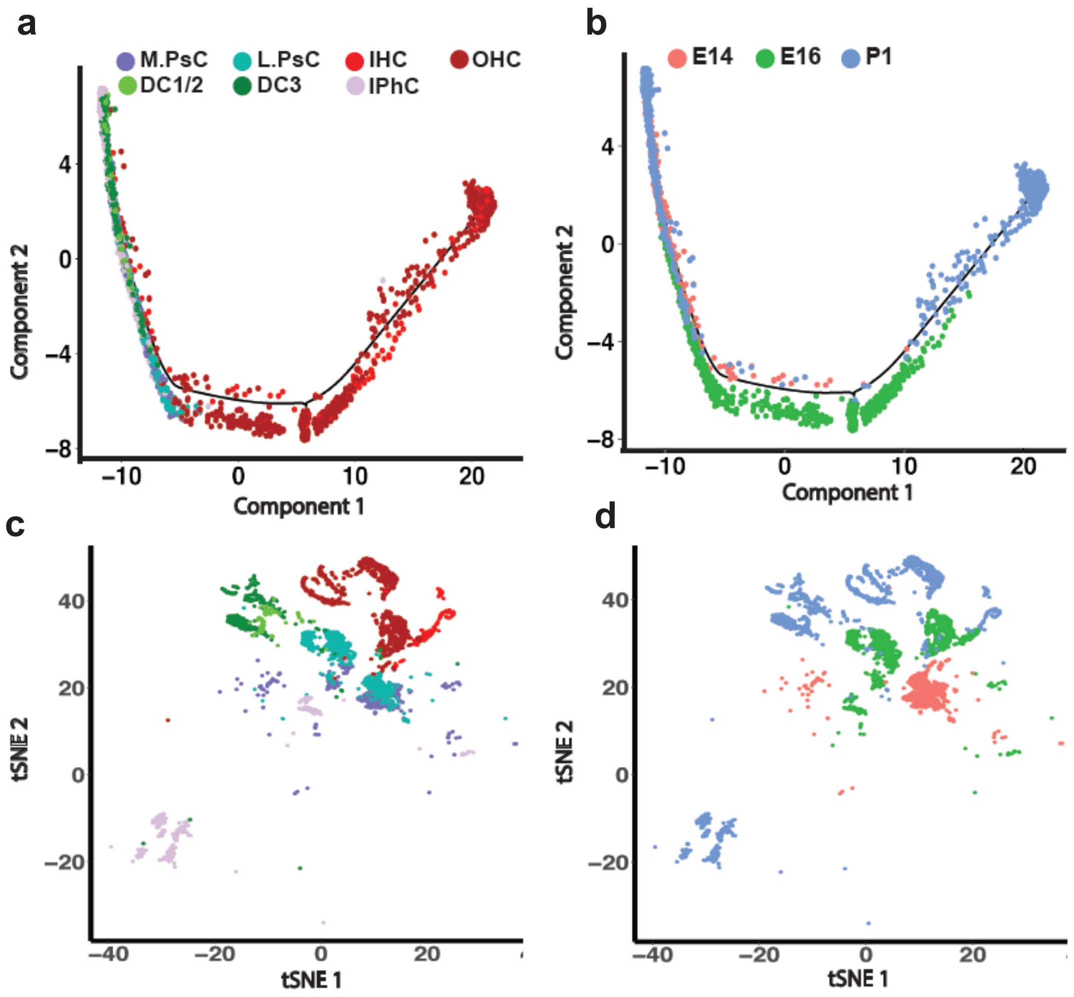

Supplementary Figure 9

Monocle trajectory for all prosensory cells, all HCs, all DCs and all IPhCs from E14, E16 and P1

a. The distribution of cell types is similar to the observed distribution for lateral cell types. Prosensory cells are located in the center of the trajectory but no clear bifurcation between HCs and SCs is present. **b.** The same trajectory indicating the age of collection for each cell. P1 cells are located towards the outer edges of each arm, consistent with their level of maturity. **c, d.** tSNE plot for the same cells as in a and b. Overall distribution indicates strong transcriptional similarities between medial and lateral prosensory cells at E14 but a greater degree of transcriptional separation at E16.

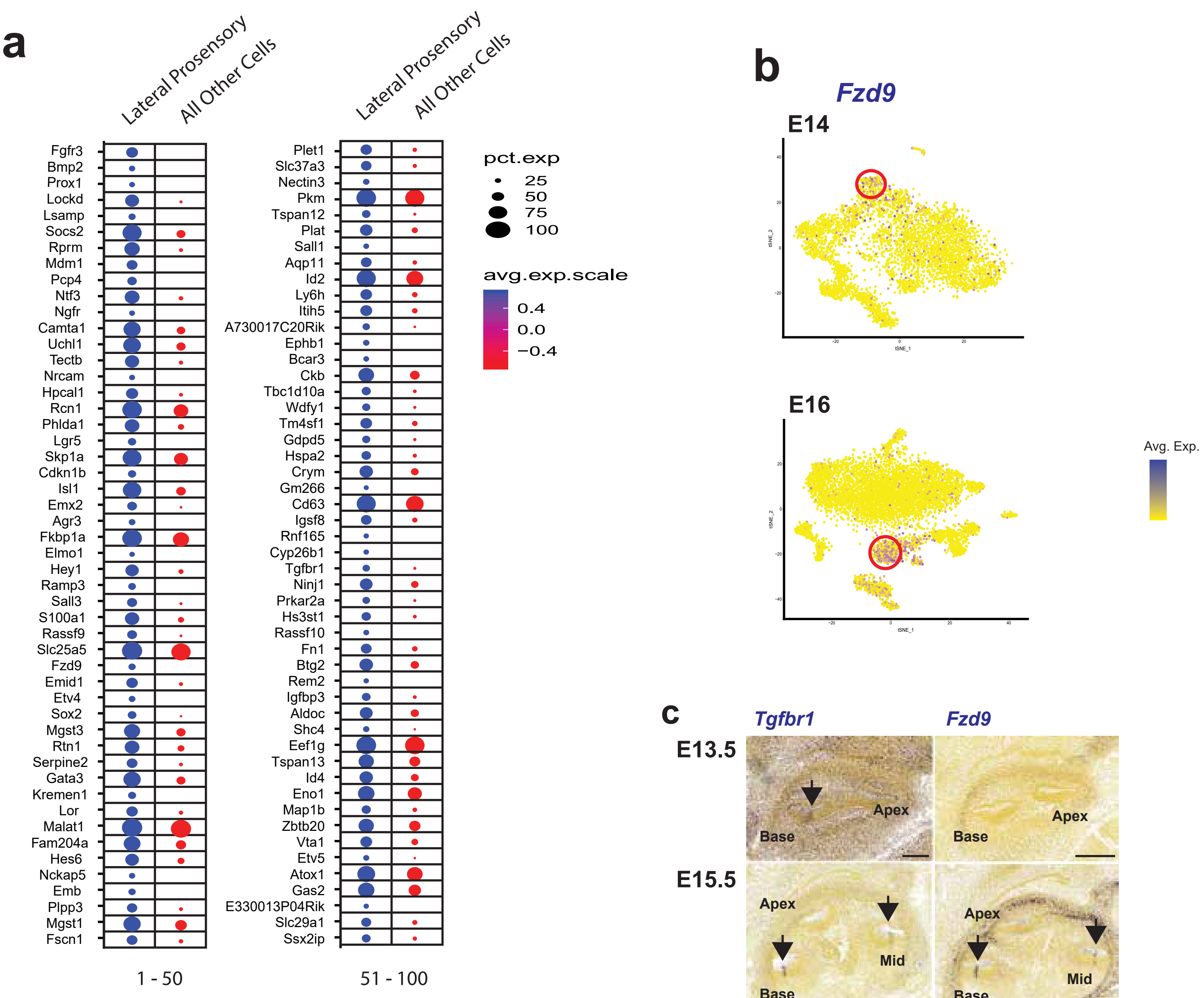

Supplementary Figure 10

Expression of lateral prosensory genes

a. Dotplot showing the level of expression for the top 100 differentially expressed genes when comparing lateral prosensory cells to all other cells at E14 (See Suppl. Data 11 for complete DE gene list). Large, blue circles indicate high expression of the listed gene in a high percentage of the lateral prosensory cells (see legend). Empty boxes indicate expression at low levels in less than 10% of cells. **b.** Feature plots for *Fzd9* in the E14 and E16 data sets. Red circles indicate lateral prosensory population. **c.** In situ hybridization images through the cochlear duct for *Tgfbr1* and *Fzd9* at E13.5 and E15.5. Arrows indicate expression in a subset of cochlear cells. Scale bars = 200 µm. Image Credit: Allen Institute, 2013 Allen Institute for Brain Science, Allen Developing Mouse Brain Atlas Tgfbr1 at E13; https://developingmouse.brain-map.org/experiment/siv?id=100053195&imageId=101048877&initImage=ish, Tgfbr1 at E15; https://developingmouse.brain-map.org/experiment/siv?id=100083731&imageId=101421542&initImage=ish, Fzd9 at E13; https://developingmouse.brain-map.org/experiment/siv?id=100040948&imageId=100853916&initImage=ish, Fzd9 at E15; https://developingmouse.brain-map.org/experiment/siv?id=100083428&imageId=101416722&initImage=ish. Number of samples with similar expression data is unclear from the Allen Brain Institute website but could be as low as one.

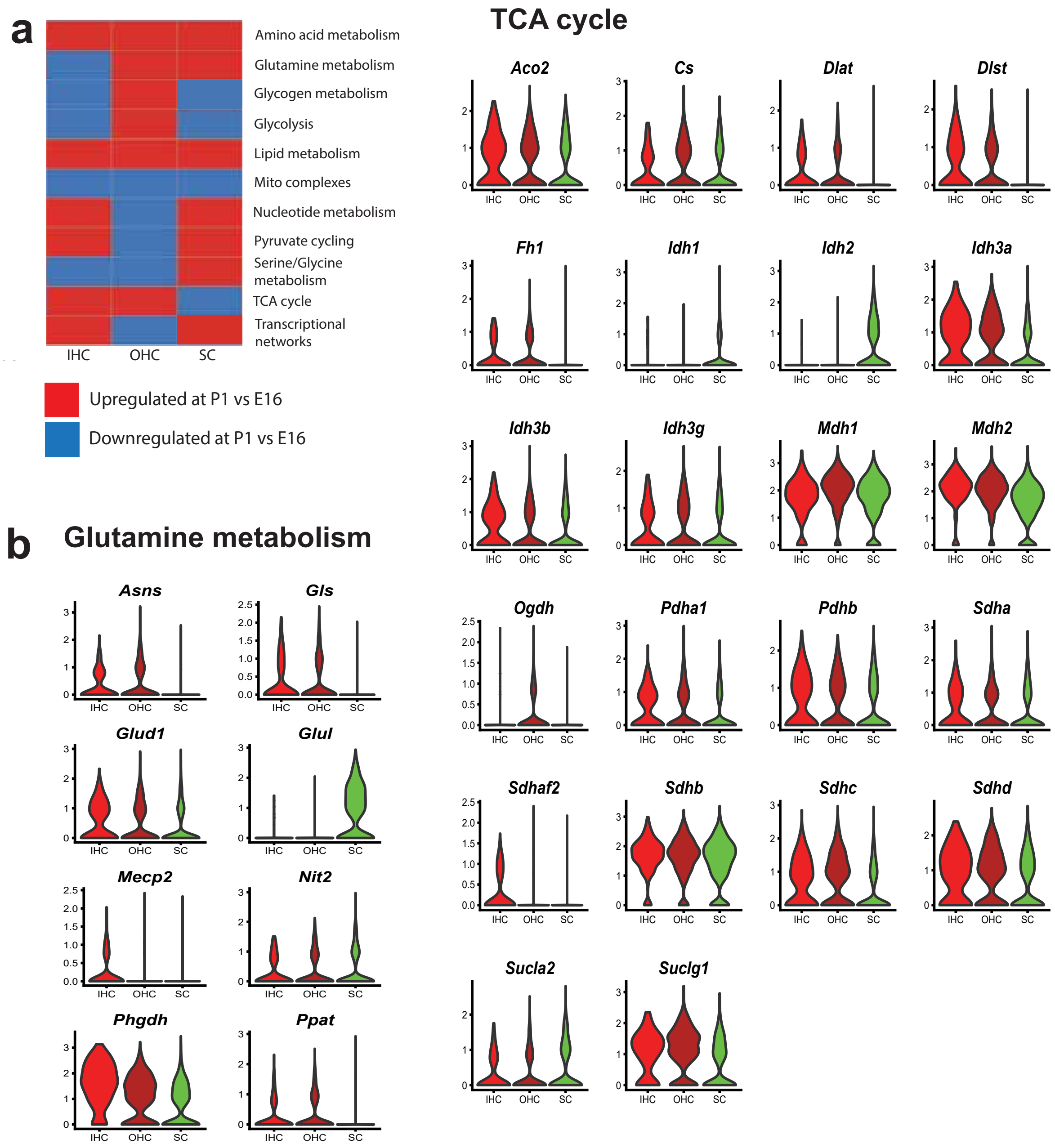

Supplementary Figure 11

Expression of metabolic genes in cochlear cells

a. Matrix illustrating predicted up (red) or down (blue) regulation of specific metabolic pathways (see methods) between the indicated cell types between E16 and P1. **b.** Violin plots for all genes within the GO gene lists for glutamine metabolism and TCA cycle that showed expression in P1 IHCs, OHCs or SCs.
