# Supplementary material for: Characterization of the development of the mouse cochlear epithelium at the single cell level
Source: Nat Commun. 2020 May 13;11:2389. doi: 10.1038/s41467-020-16113-y (PMC7221106; doi:10.1038/s41467-020-16113-y)
Supplement: Supplementary file 16 — Description of Additional Supplementary Files [file 41467_2020_16113_MOESM16_ESM.docx]

**Title: Supplementary Data 1.**

**Description:** Data on the total number of cells of each type at each age is provided. This file also provides information on the total number of cells analyzed at each time point.

**Title: Supplementary Data 2.**

**Description:** Data on differentially expressed genes in each cell type at each time point (E14, E16, P1 and P7) is provided.

**Title: Supplementary Data 3.**

**Description:** Comparison of gene expression between P1 hair cells and supporting cells collected for this study and comparable cells from Burns et al., 2015 using the gEAR compare tool.

**Title: Supplementary Data 4.**

**Description:** List of genes expressed in each phase (1-4) of P1 outer hair cell development based on trajectory analysis using Monocle

**Title: Supplementary Data 5.**

**Description:** List of genes expressed in each phase of outer hair cell development (1-4) when outer hair cells from all four time points (E14, E16, P1 and P7) are combined.

**Title:**  **Supplementary Data 6.**

**Description:** List of SCENIC regulons expressed for each cell type in the P1 dataset.

**Title: Supplementary Data 7.**

**Description:** List of SCENIC regulons expressed for each cell type in the E14 dataset.

**Title: Supplementary Data 8.**

**Description:** List of SCENIC regulons expressed for each cell type in the E16 dataset.

**Title: Supplementary Data 9.**

**Description:** List of SCENIC regulons expressed for each cell type in the P7 data set.

**Title: Supplementary Data 10.**

**Description:** List of genes expressed in each cluster (1-4) of the trajectory analysis for medial prosensory cells, inner hair cells and inner phalangeal cells.

**Title: Supplementary Data 11.**

**Description:** List of differentially expressed genes in E14 lateral prosensory cells compared with all other cells in the E14 data set.

**Title: Supplementary Data 12.**

**Description:** This file provides information on the genes within each pathway that may have contributed to metabolic shifts. Pathway analysis results in the associated figure in the main text which displays normalized frequency of upregulated genes across pathways for each condition and separately, normalized frequency of downregulated genes across pathways. This normalization accounts for geneset length and number of upregulated and downregulated genes within each geneset when comparing between the pathways.

**Title: Supplementary Data 13.**

**Description:** Comparison of differentially expressed genes between P7 inner and outer hair cells in this dataset with three previously published single cell inner and outer hair cell data sets using the gEAR comparison tool.

**Title:**  **Supplementary Data 14.**

**Description:** Number of cells captured, excluded and analyzed for each capture at each time point.
