# Supplementary material for: Characterization of the development of the mouse cochlear epithelium at the single cell level
Source: Nat Commun. 2020 May 13;11:2389. doi: 10.1038/s41467-020-16113-y (PMC7221106; doi:10.1038/s41467-020-16113-y)
Supplement: Supplementary file 18 — Reporting Summary [file 41467_2020_16113_MOESM18_ESM.pdf]

nature research

Corresponding author(s): Matthew W. Kelley

Last updated by author(s): Jan 2, 2020

Reporting Summary

Nature Research wishes to improve the reproducibility of the work that we publish. This form provides structure for consistency and transparency in reporting. For further information on Nature Research policies, see Authors & Referees and the Editorial Policy Checklist .

Statistics

For all statistical analyses, confirm that the following items are present in the figure legend, table legend, main text, or Methods section.

n/a	Confirmed	
☐	☒	The exact sample size (*n*) for each experimental group/condition, given as a discrete number and unit of measurement
☐	☒	A statement on whether measurements were taken from distinct samples or whether the same sample was measured repeatedly
☐	☒	The statistical test(s) used AND whether they are one- or two-sided<br>*Only common tests should be described solely by name; describe more complex techniques in the Methods section.*
☒	☐	A description of all covariates tested
☐	☒	A description of any assumptions or corrections, such as tests of normality and adjustment for multiple comparisons
☐	☒	A full description of the statistical parameters including central tendency (e.g. means) or other basic estimates (e.g. regression coefficient) AND variation (e.g. standard deviation) or associated estimates of uncertainty (e.g. confidence intervals)
☐	☒	For null hypothesis testing, the test statistic (e.g. *F*, *t*, *r*) with confidence intervals, effect sizes, degrees of freedom and *P* value noted<br>*Give P values as exact values whenever suitable.*
☒	☐	For Bayesian analysis, information on the choice of priors and Markov chain Monte Carlo settings
☒	☐	For hierarchical and complex designs, identification of the appropriate level for tests and full reporting of outcomes
☒	☐	Estimates of effect sizes (e.g. Cohen's *d*, Pearson's *r*), indicating how they were calculated

Our web collection on statistics for biologists contains articles on many of the points above.

Software and code

Policy information about availability of computer code

Data collection	10X Genomics Cell Ranger 2.1.1 was used to collect and initially analyze the single cell data
Data analysis	Seurat 2.0, and Monocle, R-based analysis packages for single cell RNA seq were used for these analysis of the results.

For manuscripts utilizing custom algorithms or software that are central to the research but not yet described in published literature, software must be made available to editors/reviewers. We strongly encourage code deposition in a community repository (e.g. GitHub). See the Nature Research guidelines for submitting code & software for further information.

Data

Policy information about availability of data

All manuscripts must include a data availability statement. This statement should provide the following information, where applicable:

- Accession codes, unique identifiers, or web links for publicly available datasets
- A list of figures that have associated raw data
- A description of any restrictions on data availability

All data is available both at GEO (GSE137299) and at gEAR (https://umgear.org/; permalink https://umgear.org/p?l=f7baf4ea)
Figures with associated raw data
Fig. 1,2,3,4,5,6,7

Field-specific reporting

Please select the one below that is the best fit for your research. If you are not sure, read the appropriate sections before making your selection.

Life sciences study design

All studies must disclose on these points even when the disclosure is negative.

Sample size	A minimum of three separate collections of single cells were made for each time point. For each collection, cochleae between 4 to 8 individual animals from a single litter were dissected and pooled prior to dissociation and single cell capture. These captures generated at least 4000 cells for each time point. While additional captures might decrease the variability in the data set, analysis of known cell types indicated that this number of cells was sufficient to provide a transcriptional atlas of known cell types within the cochlea.
Data exclusions	First, Seurat's "Read10x" function imported the Cell Ranger output as cell-by-gene counts expression matrices. Genes in at least 10 cells were included in the analysis. Cells with fewer than 200 unique genes and 1500 UMI or more than 3000 unique genes and 15000 UMI were excluded from the analysis. Cells with greater than 5% mitochondrial genes or greater than 5% stress genes present were excluded from downstream steps. After processing, 30,670 cells (out of 58,143) were included in the final analyzed data set (Suppl. Table 14). The expression data were then log transformed, normalized, and scaled for sequencing depth. UMI, mitochondrial content, and stress gene content scores were "regressed-out" using Seurat's "ScaleData" function. Seurat's canonical correlation analysis (CCA) accounted for batch effects between expression datasets from the same timepoint and merged these matrices to create a new object for each timepoint.
Replication	For each time point, individual cell clusters were determined. Results indicated the presence of the same cell clusters in each individual capture.
Randomization	Cells were captured in an unbiased manner and all initial cell clusters were determined using unbiased clustering in Seurat. For lineage tracing, pregnant females of the appropriate genotype were randomly selected for injections of tamoxifen or saline. For Tgfbr1 antagonist studies, explants were randomly selected for exposure to antagonist or vehicle.
Blinding	For all single cell analyses, blinding was not relevant as the initial clusters were determined using Seurat unbiased clustering. For the analysis of effects of Tgfbr1 antagonists, the researchers were not blinded but the analysis of number of outer hair cells was performed using a custom cell counting program.

Reporting for specific materials, systems and methods

We require information from authors about some types of materials, experimental systems and methods used in many studies. Here, indicate whether each material, system or method listed is relevant to your study. If you are not sure if a list item applies to your research, read the appropriate section before selecting a response.

Materials & experimental systems

n/a	Involved in the study	
☐	☒	Antibodies
☒	☐	Eukaryotic cell lines
☒	☐	Palaeontology
☐	☒	Animals and other organisms
☒	☐	Human research participants
☒	☐	Clinical data

Methods

n/a	Involved in the study	
☒	☐	ChIP-seq
☒	☐	Flow cytometry
☒	☐	MRI-based neuroimaging

Antibodies

Antibodies used	anti-FABP7 (R&D Systems), anti-CALB1 (Abcam), anti-POU4F3 (Santa Cruz Biotechnology), anti-MYO7A (Proteus Biosciences), anti-PROX1 (R&D Systems)
Validation	FABP7 https://www.rndsystems.com/search?keywords=fabp7/b-fabp site includes three citations using this antibody. Results match previously reported patterns of expression for FABP7 in the cochlea using a different antibody (Saino-Saito et al., Annals of Anatomy, 2010, 210-214<br>CALB1 https://www.abcam.com/top-739.2000122070312. Website includes four references using this anitbody<br>POU4F3 https://www.scbt.com/p/brn-3c-antibody-qq8 Website includes 15 references using this antibody<br>MYO7A http://www.proteus-biosciences.com/Product/View/Myosin-VIIa-248.aspx While website only includes 4 citations, this antibody has been used in over 100 publications looking at expression in the inner ear.<br>PROX1 https://www.rndsystems.com/products/human-prox1-antibody_af2727#product-citations website includes 54 citations using this antibody

Animals and other organisms

Policy information about studies involving animals; ARRIVE guidelines recommended for reporting animal research

Laboratory animals	CD1 and C57Bl/6 strain mice were used in these studies. Cochlea were dissected from embryos at E14 and E16, and from pups at P1 and P7.
Wild animals	*Provide details on animals observed in or captured in the field; report species, sex and age where possible. Describe how animals were caught and transported and what happened to captive animals after the study (if killed, explain why and describe method; if released, say where and when) OR state that the study did not involve wild animals.*
Field-collected samples	*For laboratory work with field-collected samples, describe all relevant parameters such as housing, maintenance, temperature, photoperiod and end-of-experiment protocol OR state that the study did not involve samples collected from the field.*
Ethics oversight	ACUC NINDS/NIDCD at NIH (Protocl 1254-18)

Note that full information on the approval of the study protocol must also be provided in the manuscript.
